# Role of the hemoglobin, albumin, lymphocyte, and platelet score in predicting thrombophlebitis among patients undergoing peripherally inserted central catheter

**DOI:** 10.1097/MD.0000000000040520

**Published:** 2024-12-06

**Authors:** Yujing Shi, Zhangli Zhan, Mengyang Ju, Ling Yang, Xiaojiao Chen, Liang Liang, Xiaolin Ge, Caiqiang Zhu, Xinchen Sun, Xiaoke Di, Chenghong He

**Affiliations:** a Department of Oncology, Jurong Hospital Affiliated to Jiangsu University, Zhenjiang, Jiangsu, China; b Department of Radiation Oncology, Osaka University, Suita, Osaka, Japan; c Department of Radiotherapy Oncology, The First Affiliated Hospital of Nanjing Medical University, Nanjing, Jiangsu, China.

**Keywords:** HALP score, peripherally inserted central catheter, thrombophlebitis

## Abstract

This study analyzes the role of the hemoglobin, albumin, lymphocyte, and platelet score (HALP), a pre-catheterization blood parameter, in predicting the occurrence of thrombophlebitis. We recruited 268 in-hospital patients who visited the Department of Oncology of our hospital and underwent peripherally inserted central catheter between January 2021 and January 2024. The cutoff value of the HALP score was defined using receiver’s operating characteristic curve, and the differences were analyzed with log-rank test. The significance of HALP in predicting thrombophlebitis was evaluated using a multivariate Cox proportional hazards model. A total of 240 patients were enrolled and divided into a high-HALP (≥31.4) group (n = 125) and a low-HALP (<31.4) group (n = 115). The relationship between the composition of HALP and clinical pathological parameters was analyzed. HALP was significantly correlated with gender (*X*^2^ = 4.74), limb restriction (*X*^2^ = 3.69), performance status score (*X*^2^ = 11.9), D-dimer (*X*^2^ = 7.88), and platelet count (*X*^2^ = 5.22). Multivariate regression analysis found male (hazard ratio [HR] 0.29 (0.12–0.69)), more puncture times (HR 0.01 (0.001–0.15)), lower HALP (HR 1.93 (0.82–4.52)), and sterile couplant (HR 20.6 (4.7–91.2)) were independent factors affecting the occurrence of thrombophlebitis. Receiver’s operating characteristic curve analysis showed the area under the curve of the HALP score was 0.718 (95% confidence interval 0.638–0.798), which was significantly larger than the other 3 parameters. Hence, we believe the predictive efficiency of HALP is higher than other parameters. The pre-catheterization HALP score can be used as a simple, accessible, and reliable tool for predicting thrombophlebitis in patients to undergo peripherally inserted central catheter.

## 1. Introduction

Peripherally inserted central catheters (PICCs) are currently the most commonly-used technology for central venous catheterization. PICCs are generally safe and convenient, and significantly outperform subcutaneous and peripheral venous catheters in patients with advanced cancers.^[1]^ In PICC, a thin catheter made of biocompatible materials (silicone or polyurethane) is inserted subcutaneously into the basilar vein or cephalic vein in the forearm or antecubital fossa under ultrasound or fluoroscopic guidance and then is advanced to the central vein. The catheter tip is usually placed in the superior vena cava or at the junction between the vena cava and the atrium.^[1]^ Compared with other central venous accesses, PICCs rarely cause complications such as hemothorax and pneumothorax.^[2]^ The most important complications of PICCs are catheter-related thrombosis and catheter occlusion, which put patients at risks and often result in additional costs in the healthcare system.^[3]^ Thrombophlebitis is a common complication of PICCs, with an incidence rate ranging from 4% to 11.7%.^[4,5]^ It refers to the presence of 2 or more of the following signs or symptoms at the catheter insertion site or adjacent veins: pain, tenderness, erythema, swelling, suppuration, palpable venous swelling, and even visible thrombosis under ultrasound.^[6–8]^

Recent studies suggest phlebitis results from external factors acting on the vein wall, including chemical stimulation, bacterial contamination and mechanical trauma. In addition, intrinsic factors including the patient’s gender, history of thrombosis, trauma, immunosuppression, diabetes, malignant tumors, high hemoglobin levels, and catheter depth also impact the occurrence of thrombophlebitis.^[9–11]^ Currently, diagnosis of thrombophlebitis is mainly based on clinical methods, including the evaluation of the patient’s symptoms and signs at the catheterization site for preliminary diagnosis. However, clinical practice shows some patients have no obvious symptoms and often require venous color Doppler ultrasound to confirm the diagnosis. If the catheter is removed without examination, there is a risk of thrombus detachment or even pulmonary embolism. However, all patients undergoing PICC must receiving venous color Doppler ultrasound before extubation, which increases the patient’s examination costs and prolongs the hospitalization time. Therefore, a simpler and more effective pre-extubation assessment indicator in clinical practice is needed to evaluate the occurrence of thrombophlebitis, conduct reasonable examinations on high-risk patients, and reduce unnecessary examination costs and the risk of thrombus detachment and even pulmonary embolism.

Nutrition and systemic inflammatory response were confirmed to be associated with tumor efficacy and survival,^[9]^ but their predictive effect on the occurrence of thrombophlebitis has never been reported. Currently, clinical predictors of nutritional and inflammatory status mainly include single hematological indicators, such as white blood cell count, platelet count, hemoglobin, and albumin, C-reactive protein. In comparison, the combination of these indicators such as neutrophil/lymphocyte ratio (NLR), platelet/lymphocyte ratio and lymphocyte/monocyte ratio can more accurately reflect the patient’s inflammatory status. The combined index HALP (hemoglobin, albumin, lymphocytes and platelets) has been recently developed and is associated with the prognosis of patients with colorectal or gastric cancer.^[10,11]^ HALP combines inflammation, nutrition and other related parameters. We believe it may be associated with the occurrence of thrombophlebitis. To date, there is no report about the effect of HALP in predicting the occurrence of thrombophlebitis in patients with PICCs. Therefore, this study was aimed to collect data from patients who had undergone PICC in the Department of Oncology of our hospital, collect hematological indicators before PICC placement, and analyze the value of pretreatment HALP parameters on predicting the occurrence of thrombophlebitis, so as to further optimize clinical practice in our hospital.

## 2. Materials and methods

### 2.1. Basic materials

A total of 268 hospitalized patients who visited the Oncology Department of our hospital and underwent PICC catheterization between January 2021 and January 2024 were randomly enrolled. The inclusion criteria were: (1) ZPS (Zubrod-ECOG-WHO performance status) score of 0–2; (2) complete medical records; (3) age at 18–80 years; (4) pathological diagnosis with malignant tumors; (5) no problem related to consciousness or sensory organs. The exclusion criteria were: (1) ZPS score > 2; (2) age > 80 years; (3) incomplete clinical data; (4) activity-caused bleeding; (5) critical infection or infection-induced shock. The chemotherapeutic drugs for all tumor-bearing patients were administered PICCs. Basic information such as previous complications, history of thrombosis, hemoglobin count, albumin count, platelet count, D-dimer, and partial prothromboplastin time before catheterization was recorded. Prior to the study, all patients were informed about the purpose of this study and were assured of their right to refuse participation or withdraw from the study at any stage. This project was approved by the Ethics Committee of Jurong People’s Hospital (approval No. JRSRMYY-2024-006).

### 2.2. Catheterization and follow-up

#### 2.2.1. Preparation before catheterization

All enrolled patients were pathologically confirmed to have malignant tumors and received intravenous treatment via PICCs. The patients were identified by the primary nurse at the title of director and above together with the attending oncologist according to the inclusion criteria. The primary nurse then assessed the patient’s vascular condition and performed puncture and catheterization. The blood vessels selected were all upper arm veins to avoid paralyzed limb and lower limb veins. Before catheterization, effective communication was conducted with the patient and family members to explain the PICC-related indications, complications, and precautions and to sign an informed consent form.

#### 2.2.2. Specific operation of PICC catheterization

Each patient was in the head-up position with the bed head raised by 15° to 30°, and the upper limb on the puncture side was abducted at 90° to the trunk. A vascular ultrasound device was correctly used to evaluate and select blood vessels, and the first choice was the basilic vein in the upper arm, followed by the brachial vein. The catheter length and arm circumference were measured as required and marked. Routine skin disinfection was performed to maximize the sterile barrier, and a drape was placed to expose the puncture site. A suitable guide pin holder was selected, and the puncture needle was installed on the ultrasound probe. The PICC catheter, the connector, and the infusion connector were pre-flushed. The assistant coated a coupling agent to the B-ultrasound probe and cooperated with the operator to cover it with a sterile plastic cover. A tourniquet was bonded to the preset puncture site: sterile conductive glue was coated, and vascular development was observed under the ultrasound machine. According to the image on the B-ultrasound screen, the probe was held by the left hand to be vertically flat against the skin. The puncture needle was held by the right hand, and the needle tip was placed with the incline upward and close to the probe. When the bright spot on the needle tip was in the center of the blood vessel and blood returned obviously in the puncture needle, it was held steadily and the probe was separated. The tip of the guide wire was inserted into the puncture needle, which was placed flat. The guide wire was sent into the vein no more than 20 cm, leaving more than 10 to 15 cm outside. The tourniquet was loosened, and the puncture needle was withdrawn. Lidocaine (0.2–0.3 mL) was used for local anesthesia around the puncture point. The skin-breaking device was placed above the guide wire, with the knife back close to the guide wire. The knife tip was pierced longitudinally into the skin by 2 to 3 mm. The vascular sheath part was passed through the guide wire, and was pushed forward into the vein from the puncture point. The guide wire and venous dilator were removed from the puncture sheath at the same time, and the sheath opening was blocked with the left thumb. The catheter was sent along the sheath opening into the blood vessel to the preset length. The tearable sheath was withdrawn from the puncture site. The catheter was fixed with one hand, and the guide wire was removed with the other hand. The catheter was trimmed to retain 5 cm of the catheter outside the body. Then decompression sleeves were installed on the catheter. A saline syringe was used to pump the returning blood and check obstruction. Then saline was injected, and the infusion connector was connected. The catheter was sealed under positive pressure, and fixed with a film. The position of the catheter tip was confirmed with X-ray photographing. According to the safe use guidelines, PICCs were not removed unless any complication or any clinical reason was found 72 to 96 hours ago.

#### 2.2.3. Follow-up and clinical diagnosis of thrombophlebitis

The included patients were followed up regularly, including physical examination, and venous color ultrasound. The follow-up was completed at June 1, 2024 for all patients. The clinical diagnosis of thrombophlebitis was made using the visual infusion phlebitis assess ment scale scoring from Kus et al.^[10]^ Visual infusion phlebitis assess ment scale is a validated visual tool that provides a numerical score based on phlebitis symptoms, such as pain, paleness, erythema, swelling, and sclerosis.

#### 2.2.4. Color ultrasound diagnosis of thrombophlebitis

**Figure 1.**
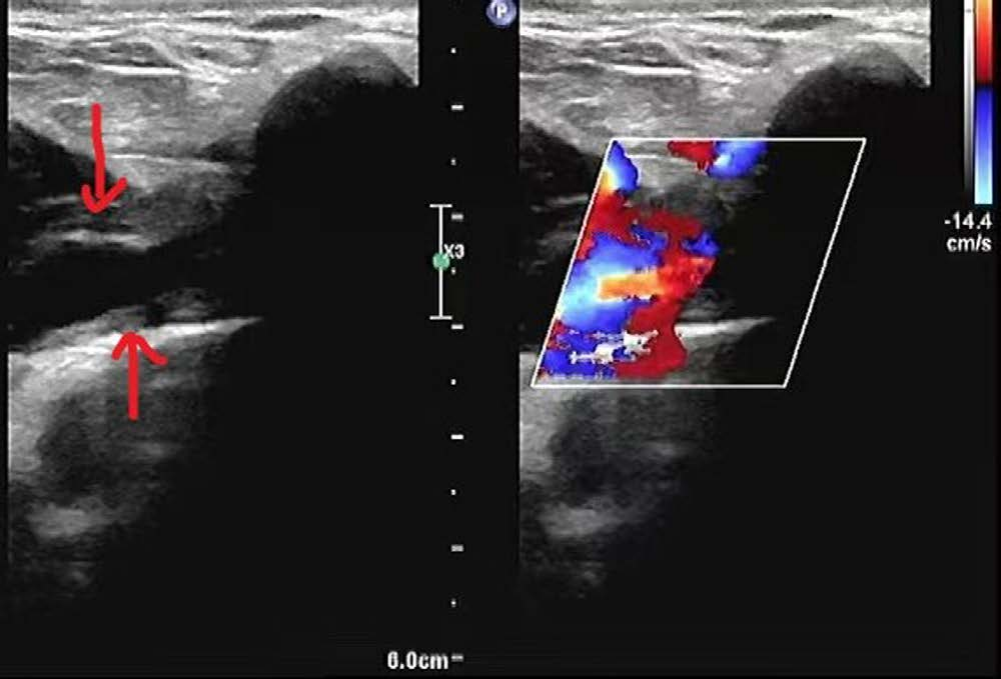
The location of venous thrombosis.

Venous color ultrasound was performed by chief radiologists or above. A Philips iU22 color Doppler ultrasound diagnosis device was used. Specifically, the probe frequency of the device was set at 10 MHZ, and then the tested patient was guided to lie in supination with the upper limbs extended and the palms facing up. The examiner placed the probe on the patient’s upper limb and performed a comprehensive exploratory ultrasound scan from bottom to top in line with the direction of blood vessels. Both parallel and cross-sectional examinations were conducted. The basilic vein, axillary vein, brachial vein, and subclavian vein of each patient were comprehensively examined. Targeted measurements of intravenous thrombus were conducted, and the anatomical structure and internal echoes of blood vessels were observed. The occurrence of thrombus in blood vessels, and the shape, size, echogenicity and attachment location of the thrombus were clarified. Color Doppler was used to observe the hemodynamics of the entire lumen, and diagnosis was made according to the criteria for thrombosis (Fig. 1).

#### 2.2.5. Definition of HALP

Hematological parameters were collected from all patients within 1 week before catheterization, including the HALP components: hemoglobin, albumin, lymphocyte count, and platelet count. HALP is computed as hemoglobin level (g/L) × albumin level (g/L) × lymphocyte level (/L)/platelet count (/L). The optimal cutoff value was determined based on the receiver’s operating characteristic (ROC) curve and the occurrence of thrombophlebitis (presence or absence). The cutoff value of HALP was 31.4, of which <31.4 was low-HALP and ≥31.4 was high-HALP.

### 2.3. Statistical analysis

Statistical analysis was conducted on SPSS 23.0 (IBM SPSS Statistics, Armonk, NY: IBM Corp) and Graphpad (GraphPad Software, Boston, MA). The influence of basic clinical factors on the composition of HALP was analyzed via χ^2^ test. The influence factors on thrombophlebitis were explored via single-factor logistic regression analysis. All factors were sent to multifactor Cox regression analysis. The hazard ratio (HR) of each factor was computed. The odds ratio, HR, and corresponding confidence interval at 95% level were determined. Unless otherwise noted, we used 2-sided significance test at α = 0.05, and *P* < .05 was considered significant.

## 3. Results

### 3.1. Baseline data

A total of 268 patients with malignant tumors were enrolled. Patients who automatically withdrew from the study during treatment and 28 patients who were discharged due to complications or sudden illness were excluded. Finally, 240 patients were enrolled, with a median age of 66 years (22–80 years). There were 116 males and 124 females. The ZPS scores were 0 to 1 in 220 patients and 2 in 20 patients. During venous color Doppler ultrasound examination, the contrast agent was normal saline in 112 patients and a sterile couplant in 128 patients. Thrombophlebitis attacked 33 of the 240 patients, including 3 patients in the normal saline group and 30 patients in the sterile couplant group. The basic information of specific clinicopathological factors is shown in Table 1.

**Table 1:** Basic information of clinical pathological factors of patients.

Characteristics	
Gender	
Male	116
Female	124
Age (years)	
Range	22–80
Median	66
ZPS score	
0–1	220
2	20
Thrombus	
Yes	3
No	237
Anticoagulant	
Yes	3
No	237
Limitation of limbs	
Yes	12
No	228
Platelet count	
≤300	194
>300	46
APTT	
≤44s	238
>44s	2
D-dimer	
≤0.5	106
>0.5	134
Puncture times	
1	235
>1	5
Indwelling time	
≤3 m	7
>3 m	233
Sterile couplant	
Yes	128
No	112
Nutrition-score (NRS2002)	
0–1	112
2–3	128

Patients’ information.

APTT = activated partial prothrombin time, ZPS = Zubrod ECOG WHO performance status.

### 3.2. Relationship between HALP and clinical pathological factors

According to the above cutoff value of HALP, we divided the enrolled patients into an H-HALP group (≥31.4, n = 125) and an L-HALP group (<31.4, n = 115). The relationship between the composition of HALP and clinical pathological parameters was analyzed. HALP was significantly correlated with gender (*X*^2^ = 4.74, *P* = .038), limb restriction (*X*^2^ = 3.69, *P* = .05), performance status score (*X*^2^ = 11.9, *P* < .001), D-dimer (*X*^2^ = 7.88, *P* = .004), platelet count (*X*^2^ = 5.22, *P* = .017) (all *P* < .05), but not with age, history of thrombosis, partial thromboplastin time, or prothrombin time (all *P* > .05) (Table 2).

**Table 2:** Relationship between HALP and clinicopathologic factors.

Characteristics	Yes (33)	No (207)	*P*
Gender			0.038^*^
Male Female	52 73	64 51	
Age (years)			0.515^*^
>65 ≤65	75 50	64 51	
ECOG-ZPS			0.000^†^
0–1 2	122 3	98 19	
Previous anticoagulation			0.53^†^
Yes No	123 2	114 1	
Platelet count			0.017^*^
>300 ≤300	108 17	86 29	
D-dimer			0.004^*^
>0.5 ≤0.5	66 59	40 75	
Limb restriction			0.05^†^
Yes No	122 3	106 9	
History of thrombosis			0.53^†^
Yes No	123 2	114 1	
APTT			0.229^†^
1	125	113	
2	1	2	
PT			0.32^*^
1 2	112 13	106 9	

APTT = activated partial prothrombin time, ZPS = Zubrod ECOG WHO performance status.

* Chi-square test.

† Fisher exact test.

### 3.3. Factors affecting thrombophlebitis

Thirty-three patients developed thrombophlebitis, including 3 patients (3/113) in the normal saline group and 30 patients (30/127) in the sterile couplant group. Then the factors affecting the occurrence of thrombophlebitis were identified via univariate regression analysis. Male gender (HR 2.4 (1.11–5.22), *P* = .026), more puncture times (HR 18.3 (3.39–98.87), *P* = .001), lower HALP score (HR 1.9 (0.82–4.52), *P* = .013), and sterile couplant (HR 20.7 (4.66–91.3) *P* = .00) were all significantly associated with the occurrence of thrombophlebitis (*P* ≤ .05). Multivariate regression analysis found male (HR 0.29 (0.12–0.69)), more puncture times (HR 0.01 (0.001–0.15)), lower HALP (HR 1.93 (0.82–4.52)), and sterile couplant (HR 20.6 (4.7–91.2)) were independent factors influencing the occurrence of thrombophlebitis (Table 3).

**Table 3:** Independent influence factors of thrombophlebitis.

Characteristics	Univariable		Multivariable	
	HR with 95% CI	*P*-value	HR with 95% CI	*P*-value
Age (years)				
≤65 >65	1.54 (0.71–3.34)	.275		
Gender				
Male Female	2.4 (1.019–5.22)	**.026**	0.28 (0.119–0.69)	**.006**
History of thrombosis				
Yes No	0.31 (0.02–3.54)	.348		
ECOG-ZPS				
0–1 2	0.607 (0.19–1.94)	.40		
Previous anticoagulation				
Yes No	0.31 (0.28–3.54)	.34		
Limb restriction				
Yes No	0.78 (0.16–3.76)	.76		
Puncture times				
1 >1	18.3 (3.38–98.8)	**.001**	0.01 (0.00–0.15)	**.001**
Lien time (months)				
<3 ≥3	0.9 (0.1–8.19)	**.967**		
Couplant				
Sterile saline	0.09 (0.02–0.304)	**.00**	20.6 (4.66–91.29)	**.000**
PT				
Normal abnormal	1.01 (0.28–3.625)	.987		
APTT				
Normal abnormal	0.15 (0.09–2.54)	.19		
PLT				
<300 ≥300	0.86 (0.34–2.13)	.748		
NLR				
>2.48 ≤2.48	1.75 (0.79–3.87)	.167		
LDH				
>193 ≤193	0.80 (0.35–1.819)	.59		
ALB				
≥37.3 <37.3	1.1 (0.47–2.64)	.79		
HALP				
<31.4 ≥31.4	0.55 (0.26–1.16)	**.012**	1.92 (0.82–4.5)	**.013**

Bold value indicates *P* ≤ .05.

ALB = albumin, APTT = activated partial prothrombin time, CI = confidence interval, LDH = lactate dehydrogenase, HALP = hemoglobin, albumin, lymphocyte, and platelet score, NLR = neutrophil lymphocyte ratio, PLT = platelet count, ZPS = Zubrod ECOG WHO performance status.

### 3.4. Efficiency of HALP

**Figure 2.**
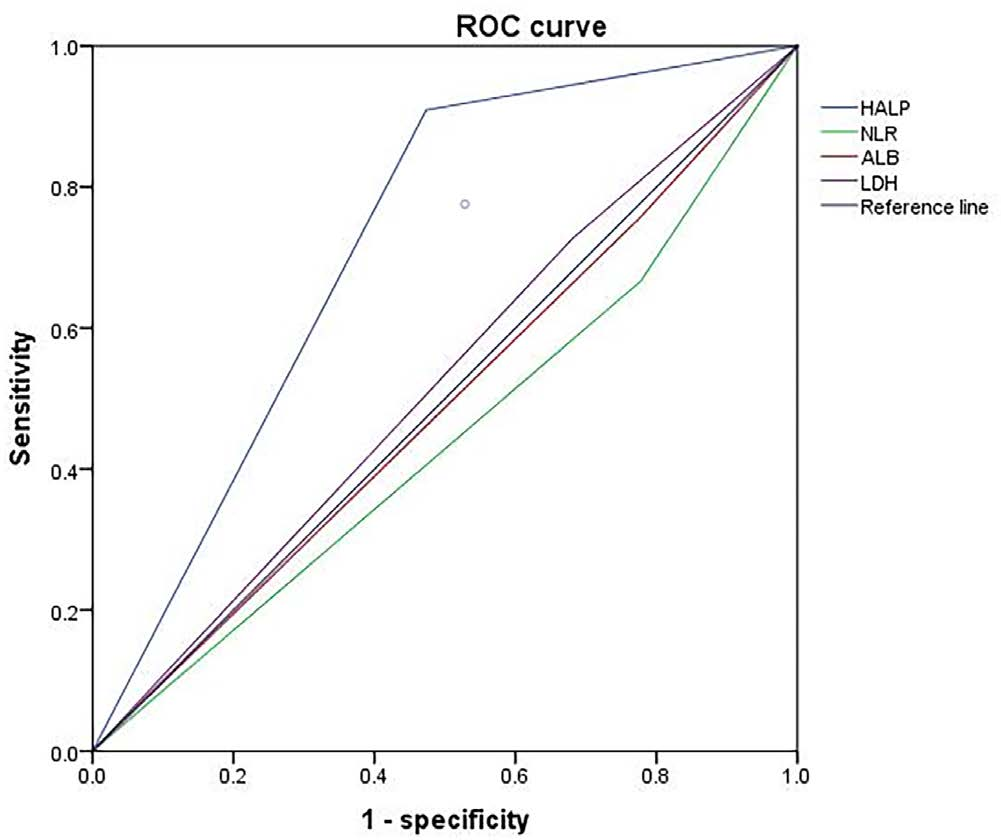
ROC curves based on the incidence of thrombophlebitis were plotted to evaluate the efficacy of HALP, ALB, NLR, and LDH. ALB = albumin, LDH = lactate dehydrogenase, HALP = hemoglobin, albumin, lymphocyte, and platelet score, NLR = neutrophil lymphocyte ratio, ROC = receiver’s operating characteristic.

At the end of follow-up, ROC curves based on the incidence of thrombophlebitis were plotted to evaluate the efficacy of HALP, albumin (ALB), NLR, and lactate dehydrogenase (LDH). In the ROC analysis, the area under curves (AUCs) of HALP score, ALB, LDH and NLR were 0.718 (95% confidence interval 0.638–0.798, *P* < .001), 0.49 (0.383–0.597, *P* = .852), 0.523 (0.42–0.63, *P* = .52), and 0.44 (0.335–0.554, *P* = .31) respectively. In summary, the AUC area of HALP is larger than that of the other 3 parameters. We believe its evaluation efficiency is better than that of the 3 parameters alone (Fig. 2).

## 4. Discussion

PICC is one of the main drug delivery methods for chemotherapy of malignant tumors. It has special characteristics, such as less trauma and easy use. Therapeutic drugs can be directly delivered to the central vein through PICC, thereby reducing the osmotic pressure and concentration of therapeutic drugs. This process also effectively avoids damage and stimulation to peripheral blood vessels, effectively reduces repeated venous punctures, and relieves pains, thus improving the quality of life of patients. PICC catheterization can be easily mastered and maintained and is safe-to-use. It significantly reduces the work and psychological pressure of nurses, and has been widely used in clinical practice.^[12–15]^ As a common venous access for cancer patients, PICC also has serious complications, such as infection, thrombosis, and displacement. Cortelezzia et al reported that the incidence of thrombosis with PICC-related symptoms in patients with malignant tumors was 1.5% to 25.7%.^[16]^ The incidence of thrombophlebitis in our study was 13.75% (33/240), which is consistent with the results of the previous study. In addition, we randomly divided the enrolled patients into 2 groups by using different venous color Doppler coupling agents and evaluated the role of sterile couplants in the diagnosis of thrombophlebitis. The incidence of thrombophlebitis in the sterile couplant group was 23.4% (30/128) and was significantly higher than in the sterile saline group (2.6%, 3/112). According to the Australian Society of Sonographers Guidelines for Safe Use and Storage of Ultrasound Couplants in Medicine,^[17]^ sterile couplants shall be used for all procedures that may contact non-intact skin or open wounds, and sterile probe covers shall be used. This study strictly followed the recommendations of the above guidelines and reached the above conclusions. Therefore, we believe the use of sterile couplants is recommended for the color Doppler ultrasound diagnosis of thrombophlebitis in order to reduce the missed diagnosis rate.

Vascular endothelial damage, blood stasis and hypercoagulable state are considered to be important causes of thrombophlebitis.^[18]^ The proceeding research shows inflammatory factors can induce the expressions of tissue factors in vascular endothelial cells, inhibit anticoagulants, induce the production of plasminogen activator inhibitors, and thus promote thrombosis.^[3]^ Therefore, we paid attention to the value of inflammatory factors in thrombophlebitis and hypothesized that clinical inflammatory markers can predict the occurrence of thrombophlebitis. In recent years, growing evidence demonstrates a strong link between systemic inflammation and tumor progression. Some inflammatory markers, such as the NLR and platelet-to-lymphocyte ratio, have showed their prognostic values in various cancers.^[19–21]^ The HALP score is a combination of inflammation, nutrition, and immune status. It was first proposed by Chen et al in 2015 to predict the survival outcomes of gastric cancer patients. Its role in the prognosis of multiple malignant tumors has been confirmed^.[22–25]^ The potential mechanism may be that the HALP score represents a combination of variables related to inflammation, nutrition, and immune status, and reflects the status of the tumor immune microenvironment in the body.^[26]^ However, there is no report on the role of HALP in the occurrence of thrombophlebitis. Therefore, we retrospectively collected the hematological parameters of 240 patients 1 week before PICC placement, tested HALP parameters, and analyzed the predictive effect of HALP on thrombophlebitis. We found HALP score (HR 1.9 (0.82–4.52), *P* = .013) was an independent factor influencing the occurrence of thrombophlebitis. We also analyzed NLR, LDH and ALB that reflect inflammation and nutrition, and found HALP had a higher value for predicting the occurrence of thrombophlebitis. The AUC of HALP score is 0.718, which is larger compared with ALB (0.49), LDH (0.523) and NLR (0.44). The AUC of HALP is 0.718, showing superior predictive performance.

To our knowledge, this study is the first to show the value of the HALP score in predicting the development of thrombophlebitis among patients receiving PICC. This study reveals several important findings. First, the HALP score is a predictor of thrombophlebitis in patients undergoing PICC. Second, the HALP score, rather than NLR, ALB, or LDH, is a useful independent prognostic factor. Finally, the HALP score is composed of common clinical hematological parameters and is convenient and accessible. Nevertheless, this study also has some limitations. First, the data came from a single-center retrospective study and may be biased. Second, the cutoff value of the HALP score was based on the median time to thrombophlebitis. The applicability of this cutoff value has not been verified and reported before, so our results need to be further confirmed by large-sample prospective studies. Third, this study only focused on the predictive effect of pre-catheterization HALP on the occurrence of thrombophlebitis and did not focus on the impact of subsequent complications and the treatment of thrombophlebitis on prognosis. Hence, further large-sample research is needed.

## Author contributions

**Conceptualization:** Yujing Shi, Mengyang Ju, Ling Yang, Xiaoke Di.

**Data curation:** Yujing Shi, Chenghong He, Mengyang Ju, Ling Yang, Liang Liang, Xiaoke Di.

**Formal analysis:** Yujing Shi, Chenghong He, Mengyang Ju, Xiaojiao Chen, Caiqiang Zhu, Xiaoke Di.

**Funding acquisition:** Chenghong He, Xiaojiao Chen.

**Investigation:** Yujing Shi, Xiaojiao Chen, Liang Liang.

**Methodology:** Yujing Shi, Ling Yang, Xiaojiao Chen, Liang Liang.

**Project administration:** Yujing Shi, Xiaojiao Chen, Xiaolin Ge.

**Resources:** Chenghong He, Xiaolin Ge.

**Software:** Yujing Shi, Caiqiang Zhu.

**Supervision:** Xiaolin Ge, Xinchen Sun.

**Validation:** Xiaolin Ge, Xinchen Sun.

**Visualization:** Ling Yang, Xiaolin Ge.

**Writing – original draft:** Yujing Shi, Mengyang Ju.

**Writing – review & editing:** Yujing Shi, Chenghong He, Xinchen Sun, Xiaoke Di.
